# Effects of different types of physical exercise interventions on aggressive behavior among children and adolescents: a network meta-analysis

**DOI:** 10.3389/fpsyg.2026.1927181

**Published:** 2026-09-09

**Authors:** Yuhang Zhang, Gyuil Lee

**Affiliations:** Department of Physical Education, Kyungpook National University, Daegu, Republic of Korea

**Keywords:** aggressive behavior, children and adolescents, network meta-analysis, physical exercise interventions, systematic review

## Abstract

**Introduction:**

Aggressive behavior is a common and important externalizing problem during childhood and adolescence, closely associated with poor social adjustment, subpar academic performance, and adverse mental health outcomes. Although physical exercise has been regarded as a viable nonpharmacological intervention to reduce aggressive behavior, the relative effects of different types of exercise interventions remain unclear. This study used a systematic review and network meta-analysis to compare how different types of physical exercise interventions affect aggression-related outcomes in children and adolescents.

**Methods:**

A systematic literature search across PubMed, Web of Science, Embase, and the China National Knowledge Infrastructure from database inception to December 10, 2025 identified randomized controlled trials (RCTs) and non-randomized controlled trials (non-RCTs) evaluating the effects of physical exercise interventions on aggression-related outcomes in children and adolescents. Based on motor skill characteristics and intervention composition, the interventions were classified into open-skill exercise (OSE), closed-skill exercise (CSE), and multicomponent exercise (MCE). Outcome measures comprised overall aggression, anger, hostility, physical aggression, and verbal aggression. Random effects models were used to conduct conventional pairwise meta-analyses and network meta-analyses, expressing effect sizes as standardized mean differences (SMDs) with 95% confidence intervals (CIs). Surface under the cumulative ranking curve (SUCRA) values determined intervention rankings, and consistency tests, meta-regression, and sensitivity analyses evaluated finding robustness.

**Results:**

This analysis included 25 studies involving 2,772 children and adolescents. Compared with control conditions, physical exercise interventions significantly mitigated negative aggression-related outcomes, including hostility (SMD = −0.37, 95% CI: −0.62 to −0.13), anger (SMD = −0.64, 95% CI: −1.00 to −0.27), overall aggression (SMD = −0.87, 95% CI: −1.36 to −0.39), and physical aggression (SMD = −0.70, 95% CI: −0.91 to −0.49). Conversely, the mitigation of verbal aggression lacked statistical significance (SMD = −0.05, 95% CI: −0.36 to 0.25). For outcomes permitting treatment network construction, the global consistency test indicated no significant inconsistency. SUCRA ranking results revealed that MCE ranked highest for hostility (79.4%) and physical aggression (74.7%), CSE ranked highest for anger (85.9%), and OSE ranked highest for overall aggression (84.4%). Meta-regression identified no significant moderators.

**Conclusion:**

Physical exercise interventions may help improve certain aggression-related outcomes in children and adolescents, although the effects are not entirely consistent across different outcomes. These findings suggest that exercise modalities possessing different skill characteristics may differ in their effects on hostility, anger, overall aggression, and physical aggression. Consequently, practitioners designing interventions for aggressive behavior in children and adolescents may consider the target outcome when selecting exercise programs. Given the substantial between-study heterogeneity and scarce head-to-head comparative evidence, additional high-quality RCTs are needed to validate these findings further.

**Systematic review registration:**

https://www.crd.york.ac.uk/PROSPERO/view/CRD42026133695, identifier (CRD420261336959).

## 1 Introduction

Aggressive behavior is one of the most common and important externalizing problems during childhood and adolescence (Hyde et al., 2026; Evans and Burke, 2024). Existing research generally conceptualizes aggression as a multidimensional construct comprising behavioral, emotional, and cognitive components. It manifests through elevated overall levels of aggression and across specific dimensions, including physical aggression, verbal aggression, anger, and hostility (Buss and Perry, 1992; Anderson and Bushman, 2002). A trend study utilizing data from 190,493 adolescents across 30 countries in the Global School-based Student Health Survey reported mean 12-month prevalence rates of physical fighting and physical attacks at 36.5 and 37.2%, respectively (Smith et al., 2024). Such problems directly disrupt peer relationships, classroom order, and school adjustment, while correlating closely with depression, loneliness, difficulties in emotion regulation, and other adverse psychosocial outcomes (Ladd and Burgess, 1999; Estévez et al., 2018; Smith et al., 2026). More importantly, elevated aggression levels during adolescence exhibit continuity over time; left unaddressed, these behaviors elevate the risk of antisocial personality disorder and violent behavior in adulthood (Loeber and Hay, 1997; Whipp et al., 2019; Fite et al., 2009). Therefore, early identification and effective interventions targeting multidimensional aggression-related outcomes in children and adolescents carry clear importance for educational practice and public health (Jiang et al., 2024; Bierman et al., 2025; Hyde et al., 2026).

Among available intervention strategies, physical exercise attracts considerable attention because of its potential to promote emotion regulation, self-control, and self-regulation (Fuentealba-Urra et al., 2023; D'Cruz et al., 2024). Previous studies have shown that regular participation in physical exercise improves mental health in children and adolescents, while positively influencing aggression-related outcomes by providing an appropriate outlet for emotional release, enhancing self-control, and promoting prosocial behavior (Singh et al., 2025; Mao et al., 2024; Wade et al., 2026). Physical exercise thus represents a feasible approach for addressing aggression-related problems in children and adolescents. Existing reviews have also suggested that exercise interventions may help reduce aggression levels in this population (Zhao and Wang, 2025). However, these effects vary across different dimensions of aggression, and factors such as exercise type and intervention format may further alter intervention effectiveness (Ouyang and Liu, 2023; Wang et al., 2025; Wang et al., 2025). More importantly, existing reviews have primarily focused on the overall effects of exercise interventions on aggression-related outcomes. Although some studies have begun to examine differences across exercise types and intervention characteristics, these analyses have still relied mainly on moderator analyses or subgroup comparisons within traditional meta-analyses (Zhu F. et al., 2022; Zhu Y. et al., 2022; Yang et al., 2023). Therefore, current evidence fails to systematically compare the relative advantages of different exercise types across various aggression-related outcomes in children and adolescents within a unified framework, limiting deeper understanding of the relationship between specific exercise characteristics and intervention outcomes.

Different exercise modalities differ substantially in environmental characteristics and information-processing demands (Purgato et al., 2024; Heilmann et al., 2022). The occurrence and regulation of aggressive behavior involve not only emotional reactivity but also inhibitory control, self-regulation, and information-processing mechanisms within social contexts. Therefore, classifying exercise modalities according to their differing cognitive and behavioral regulatory demands may provide a useful framework for understanding potential effects on aggression-related outcomes (Smeijers et al., 2020; Feng et al., 2023). Based on the predictability of the exercise environment, exercise modalities are typically classified as open-skill exercise (OSE) and closed-skill exercise (CSE) (Lai et al., 2024). OSE is typically performed in dynamically changing environments and requires individuals to continuously allocate attention, inhibit responses, and make rapid decisions in response to external stimuli. For example, soccer and volleyball require participants to continuously adjust their movements in response to changes in the positions and movements of teammates, opponents, and the ball. By contrast, CSE is usually performed in relatively stable and predictable environments, allowing individuals to plan their movements more fully in advance, with comparatively lower demands on real-time information processing. Activities such as yoga and running, for instance, generally involve relatively stable environmental conditions and repetitive or preplanned movement patterns. (Feng et al., 2023; Wang et al., 2025; Wang et al., 2025). However, exercise formats used in real-world interventions exhibit greater complexity. School-based programs frequently combine skill practice, cooperative tasks, game-based activities, and physical fitness training, thereby incorporating open-skill demands and closed-skill components. Such interventions are difficult to classify into a single category and warrant consideration as multicomponent exercise (MCE) (Liu H. et al., 2025, Liu C. et al., 2025). This classification accurately reflects the complexity of real-world interventions and establishes a clear analytical framework to compare the relative effects of different exercise modalities.

However, existing evidence comparing the relative effects of different skill-based exercise modalities remains limited. Although conventional pairwise meta-analysis can evaluate the overall effects of exercise interventions on aggression-related outcomes in children and adolescents, systematically comparing the relative advantages of different exercise modalities remains difficult when head-to-head evidence is lacking (Brignardello-Petersen and Guyatt, 2025). Conversely, network meta-analysis can integrate direct and indirect evidence within a single framework to compare the relative effects and rankings of multiple interventions (Chaimani et al., 2024). Therefore, the present study conducted a systematic review and network meta-analysis. Based on exercise context, movement characteristics, and the combined formats used in real-world applications, exercise interventions were classified into three categories: OSE, CSE, and MCE. The investigation aims to compare the relative effects of these modalities on aggression-related outcomes in children and adolescents, providing empirical evidence to inform the selection of intervention strategies. Specifically, it addresses the following questions: (1) whether physical exercise interventions reduce hostility, anger, overall aggression, physical aggression, and verbal aggression compared with control conditions; and (2) whether OSE, CSE, and MCE differ in their relative effects across these aggression-related outcomes.

## 2 Methods

The International Prospective Register of Systematic Reviews (Registration No. CRD420261336959) registered this study protocol (PROSPERO record: https://www.crd.york.ac.uk/PROSPERO/view/CRD420261336959). The execution and reporting of this systematic review and network meta-analysis adhered to the Preferred Reporting Items for Systematic Reviews and Meta-Analyses (PRISMA) guidelines (Page et al., 2021). (see Supplementary Material 2).

### 2.1 Inclusion and exclusion criteria

The inclusion and exclusion criteria were established according to the PICOS framework. (a) Participants were children and adolescents aged 6–20 years (Viner and Barker, 2005); studies involving adults, older adults, infants, or populations with unclear demographic information were excluded. (b) Interventions consisted of structured exercise programs, including OSE, CSE, or MCE; studies evaluating unstructured physical activity, single-session interventions, insufficient intervention duration, or poorly described training characteristics were excluded. (c) Control conditions included participants receiving no additional exercise intervention, those receiving unstructured activities, or those undergoing alternative exercise modalities, which indicates that studies lacking a control group or displaying significant baseline differences were excluded. (d) Outcome assessment required standardized, validated instruments measuring aggression-related outcomes, including overall aggression, physical aggression, verbal aggression, hostility, and anger; research omitting relevant outcome data, providing incomplete statistical information, withholding original data upon request was excluded. (e) Eligible study designs included randomized controlled trials (RCTs) and non-RCTs (nRCTs) that provided clear between-group comparisons and sufficient baseline data. Studies using subjective allocation procedures, single-arm designs, cohort structures, or cross-sectional frameworks were excluded, and conference abstracts, gray literature, dissertations, and studies without full-text availability were likewise excluded.

### 2.2 Search strategy

A systematic literature search was conducted across four major databases: PubMed, Web of Science, Embase, and the China National Knowledge Infrastructure. These databases were selected to ensure comprehensive coverage of authoritative, current research in medicine, exercise science, and mental health. Incorporating a leading Chinese database further enhanced search completeness. The search spanned from database inception to December 10, 2025. The search terms covered children and adolescents, physical exercise and sports interventions, and aggression-related outcomes. The complete database-specific search strategies are provided in Supplementary Data 1.

### 2.3 Study selection and data extraction

Study selection followed the PRISMA guidelines. First, all records retrieved from the database searches were imported into EndNote 21, and duplicate records were removed through a combination of automated deduplication and manual verification. Subsequently, two independent reviewers (YZ and YS) screened the remaining citations in sequential stages based on the prespecified eligibility criteria. Title and abstract screening first eliminated clearly irrelevant records, followed by full-text retrieval and evaluation to determine final study inclusion. Discussion between the two reviewers resolved any screening disagreements, and a third reviewer (Lee) provided adjudication when consensus was unreachable.

A predesigned, standardized form facilitated independent data extraction by the two reviewers, who cross-checked all extracted parameters for accuracy. Extracted data comprised baseline study characteristics (authors, publication year, and region), participant demographics (sample size, age, and sex), intervention parameters (exercise type, intervention duration, session length, and frequency), and outcome metrics (measurement instruments, and pre- and post-intervention means and standard deviations). To facilitate comparisons across studies, exercise interventions were classified into three categories based on the exercise environment, motor skill characteristics, and intervention composition: (1) OSE, performed in dynamic environments characterized by changing external conditions and requiring continuous adaptation to external stimuli; (2) CSE, performed in stable and predictable environments, with movements that can generally be planned in advance; and (3) MCE, comprising programs that integrated open- and closed-skill elements or multiple distinct exercise components. Based on this classification, each eligible comparison was assigned to the appropriate outcome-specific synthesis according to the intervention category, comparator, and reported aggression-related outcome. All eligible aggression-related outcomes, including overall aggression, physical aggression, verbal aggression, hostility, and anger, were extracted and analyzed separately. When a study reported both an overall aggression score and scores for specific aggression dimensions, these data were extracted separately and included in the corresponding outcome-specific analyses. When multiple instruments assessed the same outcome domain, the measure designated as primary in the original study was selected. When multiple assessment time points were reported, data from the first post-intervention assessment were used, and subsequent follow-up data were excluded. The formulas were as follows (Follmann et al., 1992):

$$M=M_{2}-M_{1}$$

$$\mathrm{SD}=\sqrt{SD_{1}^{2}+SD_{2}^{2}-2\times R\times SD_{1}\times SD_{2}}$$

M, mean change score; M₁, pretest mean; M₂, posttest mean; SD, standard deviation of change scores; SD₁, pretest standard deviation; SD₂, posttest standard deviation; *r*, pre–post correlation coefficient, set at 0.50.

Discrepancies or data ambiguities required original article consultation, mutual discussion, or third-reviewer adjudication. When necessary, electronic correspondence with the corresponding authors generated the missing data. If the required data could not be obtained, the study was excluded from the relevant analysis.

### 2.4 Risk of Bias assessment and confidence in network Meta-analysis (CINeMA)

Two reviewers independently assessed the risk of bias in the included studies using the Cochrane Risk of Bias tool (Higgins et al., 2011) within RevMan 5.4 (The Cochrane Collaboration, 2020). Any disagreements were resolved through discussion, with a third reviewer consulted when necessary. Evaluated domains included random sequence generation, allocation concealment, blinding of participants and personnel, blinding of outcome assessment, completeness of outcome data, selective reporting, and supplementary bias sources. Each domain was rated as “low risk,” “high risk,” or “unclear risk” (Higgins et al., 2011). The certainty of the network evidence was evaluated using the CINeMA framework across six domains: within-study bias, reporting bias, indirectness, imprecision, heterogeneity, and incoherence (Nikolakopoulou et al., 2020). Two reviewers independently conducted the CINeMA assessments, resolving disagreements through discussion and consulting a third reviewer when consensus could not be reached. Publication bias was assessed using funnel plots and Egger’s test (Egger et al., 1997). Regarding imprecision, a standardized mean difference (SMD) of 0.20 represented the minimal important effect; the evidence certainty was downgraded if the 95% confidence interval (CI) spanned across the range from −0.20 to 0.20 (Schünemann et al., 2022). The evidence was initially rated as high and downgraded according to the assessments across the six domains. A rating of “some concerns” in a domain generally resulted in a one-level downgrade, whereas “major concerns” generally resulted in a two-level downgrade. The final evidence rating was determined by considering all six domains jointly: no downgrade, a one-level downgrade, a two-level downgrade, and a downgrade of three or more levels corresponded to high, moderate, low, and very low, respectively (Nikolakopoulou et al., 2020).

### 2.5 Statistical analysis

All statistical analyses were performed using Stata 18.0 (StataCorp, 2023). For each outcome reported in at least two studies, a conventional meta-analysis using a random effects model pooled the effect sizes, expressing intervention effects as SMDs and corresponding 95% Cis (Deeks et al., 2024; Higgins et al., 2024). In accordance with the Cochrane Handbook, SMD magnitudes were interpreted as small (SMD < 0.40), moderate (0.40–0.70), or large (>0.70) (Schünemann et al., 2024). Between-study heterogeneity was assessed using Cochran’s Q test and the *I^2^* statistic, with *I^2^* values of 25, 50, and 75% indicating low, moderate, and high heterogeneity, respectively (Cochran, 1954; Higgins et al., 2003). For outcomes featuring at least 10 studies and showing substantial heterogeneity (*I^2^* > 50%), meta-regression analyses were further conducted to explore potential heterogeneity sources using sample size, mean age, intervention duration, and intervention frequency as covariates (Higgins and Thompson, 2004). The 10-study threshold was selected because meta-regression with fewer than 10 studies may have limited statistical power and yield unreliable results (Deeks et al., 2024). In addition, leave-one-out sensitivity analyses were performed for outcomes with substantial heterogeneity by sequentially excluding each study and recalculating the pooled effect estimate to assess the robustness of the findings. Moreover, subgroup analyses were conducted according to exercise format (team sports vs. individual sports).

Network meta-analysis extends conventional pairwise meta-analysis by combining direct and indirect evidence within a connected evidence network, thereby allowing multiple interventions, including those that have not been directly compared, to be compared simultaneously. On this basis, a network meta-analysis was further conducted within a frequentist framework using the network package in Stata 18.0 to compare and rank the relative effects of different exercise interventions (White, 2015; Supplementary Data 2). A network plot was initially constructed to illustrate the structure of the evidence network, with nodes representing the interventions and lines connecting the nodes representing direct comparisons (Chaimani et al., 2013). Local inconsistency was assessed using the node-splitting method, which compares direct and indirect effect estimates. When no statistically significant difference was found between the two estimates (*p* > 0.05), the corresponding comparison was considered to show no evidence of local inconsistency. The surface under the cumulative ranking curve (SUCRA) was used to summarize the ranking probabilities of the interventions, with higher values indicating a greater probability of achieving a favorable rank. Rank plots were used to visualize the ranking results. Study characteristics and synthesis results were presented in tables, including a league table. Additional figures, including forest plots from the pairwise meta-analyses, were provided in Supplementary Material 1.

### 2.6 Publication bias

To assess potential publication bias in the present study, funnel plots were initially constructed to provide a preliminary visual inspection of the symmetry of study distribution, followed by Egger’s linear regression test for quantitative statistical assessment (Egger et al., 1997). For outcomes suggesting possible publication bias, the trim-and-fill method was further applied (Duval and Tweedie, 2000).

## 3 Results

### 3.1 Included studies and their characteristics

**Figure 1:**
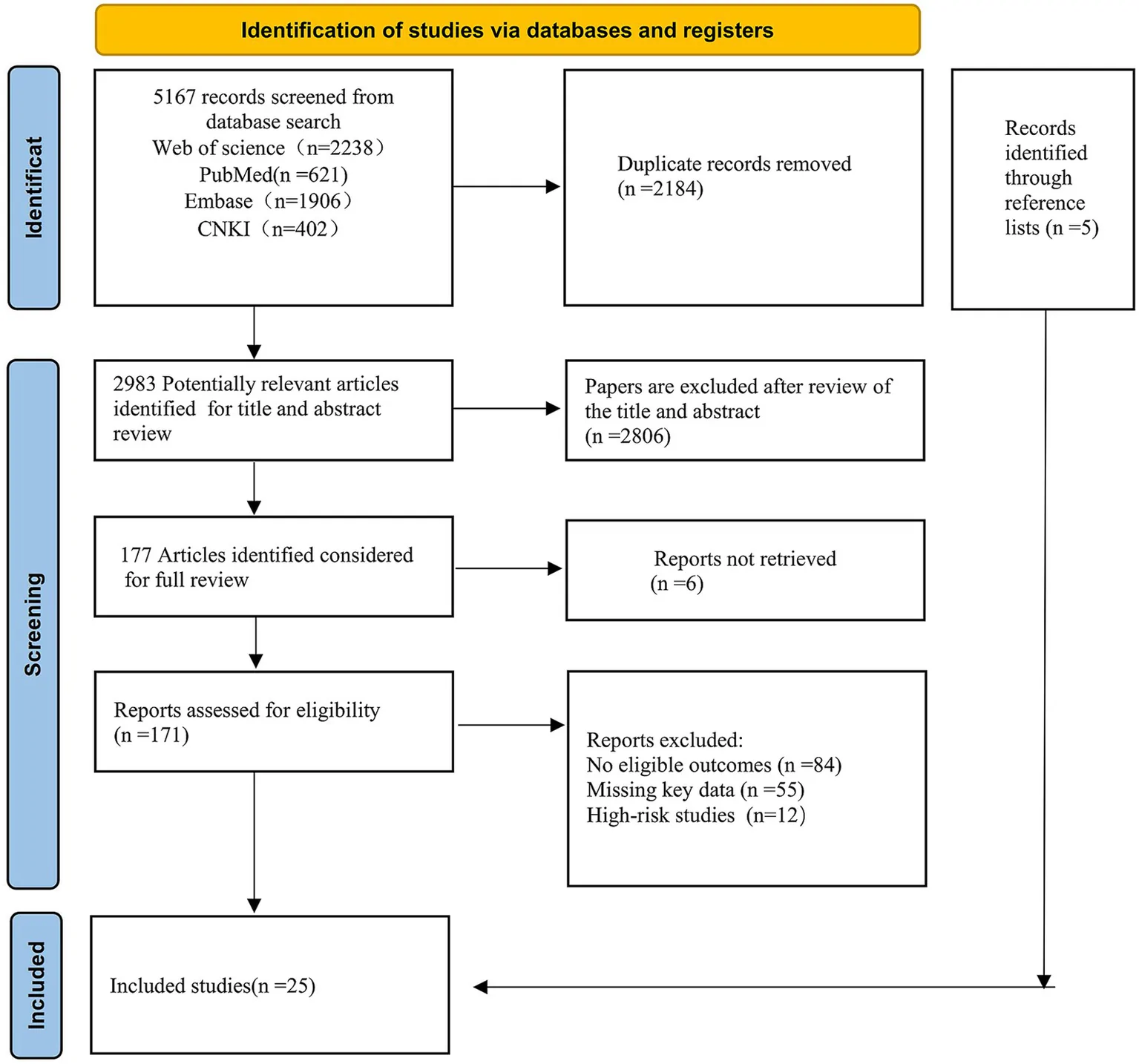
Flowchart of the selection process.

The systematic search identified 5,167 potentially eligible records, and manual searching plus citation tracking retrieved 5 supplementary entries. After duplicate removal, 2,983 records proceeded to title and abstract screening. Title and abstract evaluation eliminated 2,806 records, leaving 177 full-text articles for explicit eligibility assessment. According to the predefined inclusion and exclusion criteria, 25 studies ultimately met the inclusion requirements for the final synthesis, tracking a total of 2,772 children and adolescents (Butzer et al., 2017; Chen and Diao, 2007; Fung and Lee, 2018; Guo et al., 2025; Haden et al., 2014; Harwood-Gross et al., 2021; He, 2007; Liang et al., 2024; Liu H. et al., 2025, Liu C. et al., 2025; Lok et al., 2024; Mehralian et al., 2022; Park et al., 2017; Parsamajd and Teymori, 2024; Perić et al., 2022; Reynes and Lorant, 2002; Shachar et al., 2016; Tkacz et al., 2008; Trajković et al., 2020a, 2020b; Velásquez et al., 2015; Wade et al., 2018; Yang et al., 2018; Zhao et al., 2020; Zhu and Yan, 2006; Zhu F. et al., 2022; Zhu Y. et al., 2022). Figure 1 illustrates the study selection process, and Table 1 summarizes the baseline characteristics of the included research.

**Table 1:** Basic characteristics of the included studies.

Author	Country	Age	Sample		Intervention		Exercise type	Measurement	Intervention dose	Outcome
			T	C	T	C				
Zhao et al., 2020	China	6–15	10	10	OSE	MCE	Volleyball VS Baduanjin	BPAQ	3 × 30-min sessions/week for 8 weeks	①②④⑤
Trajković et al., 2020a	Serbia	T:15.7 ± 0.6C:15.8 ± 0.5	54	51	OSE	N	soccer	BPAQ	2 × 45-min sessions/week for 35 weeks	①②④⑤
Yang et al., 2018	South Korea	T:11.6 ± 0.5C:11.6 ± 0.5	8	8	OSE	N	Taekwondo	K-POMS	1 × 60-min session/week for 16 weeks	②
Park et al., 2017	South Korea	T:11.60 ± 0.84C:12.10 ± 0.56	23	25	MCE	N	Physical education curriculum	Aggression Scale	2 × 50-min sessions/week for 8 weeks	③
Mehralian et al., 2022	Iran	7–10	15	15	CSE	N	Yoga	CBCL	2 × 60-min sessions/week for 10 weeks	③
Parsamajd and Teymori, 2024	Iran	9–12	38	38	OSE	N	Karate	CBCL	3 × 60-min sessions/week for 12 weeks	③
Liu H. et al., 2025, Liu C. et al., 2025	HK, China	T:15.82 ± 1.11C:13.65 ± 1.21	40	40	CSE	N	aerobic exercise	BPAQ	1 × 60-min session/week for 12 weeks	③
Liang et al., 2024	China	T:8.42 ± 1.12C:8.82 ± 1.59	15	15	MCE	N	Multicomponent MVPA	CBCL	3 × 60-min sessions/week for 12 weeks	③
He, 2007	China	17–19	60	60	MCE	N	Basketball, Volleyball, Aerobics	POMS	6 × 60-min sessions/week for 13 weeks	②
Trajković et al., 2020b	Serbia	T:15.5 ± 0.7C:15.7 ± 0.6	56	51	OSE	N	Volleyball	BPAQ	2 × 45-min sessions/week for 35 weeks	①②④⑤
Shachar et al., 2016	Israel	8–12	330	319	MCE	N	Martial arts, group sports	BPAQ	5 × 50-min sessions/week for 24 weeks	①②④
Harwood-Gross et al., 2021	Israel	T:15.8 ± 0.78C:15.4 ± 0.78	20	19	OSE	N	Martial arts	Aggression Scale	2 × 50-min sessions/week for 26 weeks	③
Haden et al., 2014	USA	10–11	15	15	CSE	N	Yoga	CBCL	3 × 90-min sessions/week for 12 weeks	③
Chen and Diao, 2007	China	T:21.2 ± 1.05C:20.4 ± 1.34	30	30	CSE	N	Aerobics	POMS	3 × 60-min sessions/week for 8 weeks	②
Tkacz et al., 2008	USA	T:9.7 ± 1.1C:9.8 ± 1.1	139	69	CSE	N	Aerobic exercise	PAES	5 × 60-min sessions/week for 13 weeks	②
Lok et al., 2024	Turkey	T:15.12 ± 0.75C:15.96 ± 0.79	40	40	CSE	N	Aerobic exercise	AARS	4 × 60-min sessions/week for 10 weeks	②
Wade et al., 2018	Australia	T:12.7 ± 0.5C:12.7 ± 0.5	137	152	MCE	N	Multicomponent physical activity program	Aggression Scale	2 × 30-min sessions/week for 35 weeks	③
Fung and Lee, 2018	HK, China	T:8.53 ± 1.11C:8.57 ± 1.11	75	67	OSE	CSE	Martial arts VS Physical fitness training	RPQ	1 × 90-min session/week for 10 weeks	③
Zhu and Yan, 2006	China	T:20.05 ± 1.22C:20.13 ± 1.18	29	30	OSE	N	Basketball	SCL-90	3 × 30-min sessions/week for 10 weeks	①
Reynes and Lorant, 2002	France	8	28	27	OSE	N	Judo	BPAQ	2 × 90-min sessions/week for 52 weeks	①②④⑤
Perić et al., 2022	Serbia	T:15.7 ± 0.5C:15.7 ± 0.5	12	13	OSE	N	Soccer	CBCL	2 × 60-min sessions/week for 16 weeks	③
Butzer et al., 2017	USA	T:12.64 ± 0.33C:12.64 ± 0.33	116	93	CSE	N	Yoga	BRUMS	2 × 35-min sessions/week for 26 weeks	②
Velásquez et al., 2015	Colombia	10–15	68	57	CSE	N	Yoga	PNIA	2 × 120-min sessions/week for 12 weeks	③
Guo et al., 2025	China	9	45	45	OSE	N	Soccer	CABQ	3 × 60-min sessions/week for 13 weeks	③
Zhu F. et al., 2022; Zhu Y. et al., 2022	China	17–19	37	43	OSE	N	Basketball	BPAQ	2 × 30-min sessions/week for 12 weeks	①②④⑤

In the sample size, T denotes the intervention group and C denotes the control group. Measurement tools: BPAQ, Buss–Perry Aggression Questionnaire; K-POMS, Korean version of the Profile of Mood States; Aggression Scale; CBCL, Child Behavior Checklist; POMS, Profile of Mood States; PAES, Pediatric Anger Expression Scale; AARS, Adolescent Anger Rating Scale; RPQ, Reactive-Proactive Aggression Questionnaire; SCL-90, Symptom Checklist-90; BRUMS, Brunel Mood Scale; PNIA, Peer-Nominated Index of Aggression; CABQ, Classroom Aggressive Behavior Questionnaire. The outcome measures are as follows: ① hostility, ② anger, ③overall aggression, ④ physical aggression, and ⑤ verbal aggression.

The included studies spanned multiple countries and regions, featuring 9 from China (comprising 2 from Hong Kong); 3 each from the United States and Serbia; 2 each from South Korea, Iran, and Israel; and 1 each from Australia, France, Turkey, and Colombia. Regarding specific variables, 12 studies reported overall aggression, 12 measured anger, 7 evaluated hostility, 6 documented physical aggression, and 5 detailed verbal aggression. Based on the cognitive demands associated with motor skill execution, the evaluated treatments fell into three categories: OSE, CSE, and MCE.

### 3.2 Risk of bias assessment and CINeMA

**Figure 2:**
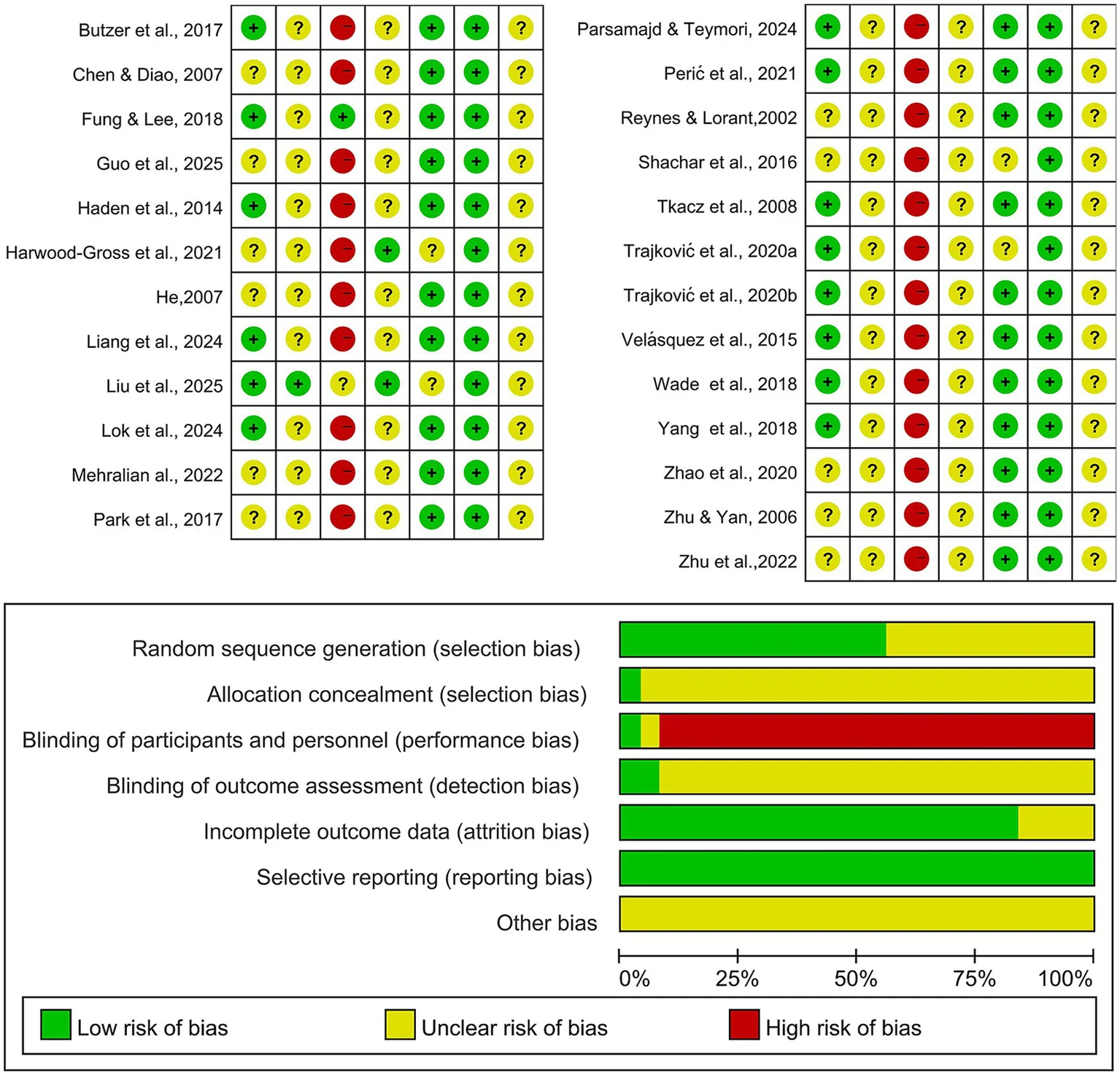
Graph of Cochrane risk bias assessment.

Figure 2 presents the methodological quality evaluations generated via the Cochrane Risk of Bias tool. The two reviewers disagreed on the random sequence generation domain for Zhao et al. (2020). After discussion, the domain was rated as unclear risk without third-reviewer adjudication. Random sequence generation and completeness of outcome data generally exhibited a low risk of bias across the literature. By contrast, allocation concealment, blinding of outcome assessment, and supplementary bias sources mostly received unclear risk ratings, whereas blinding of participants and personnel predominantly presented a high risk of bias. Selective reporting bias represented a low risk across all included studies.

Two study-specific ratings warrant further clarification. In Liu H. et al., (2025); Liu C. et al., (2025), allocation concealment was rated as low risk because computer-generated block randomization was conducted by an independent research assistant, and the allocation sequence remained concealed until intervention assignment. However, blinding of participants and personnel was rated as unclear risk because participant blinding was not explicitly reported. In Fung and Lee (2018), blinding of participants and personnel was rated as low risk. This rating was primarily based on the use of a cluster-randomized design, in which 31 clusters were randomly assigned to structured intervention conditions using a blinded allocation procedure, with physical fitness training serving as a placebo control rather than a no-intervention control.

The certainty of the evidence for the network comparisons was assessed using the CINeMA framework. Among the 14 network comparisons, seven were rated as having low certainty and seven as having very low certainty. The main reasons for downgrading were concerns regarding within-study bias in all 14 comparisons, imprecision in eight comparisons, and heterogeneity in ten comparisons. Detailed comparison-specific assessments are presented in Supplementary Table S1.

### 3.3 Hostility

**Figure 3:**
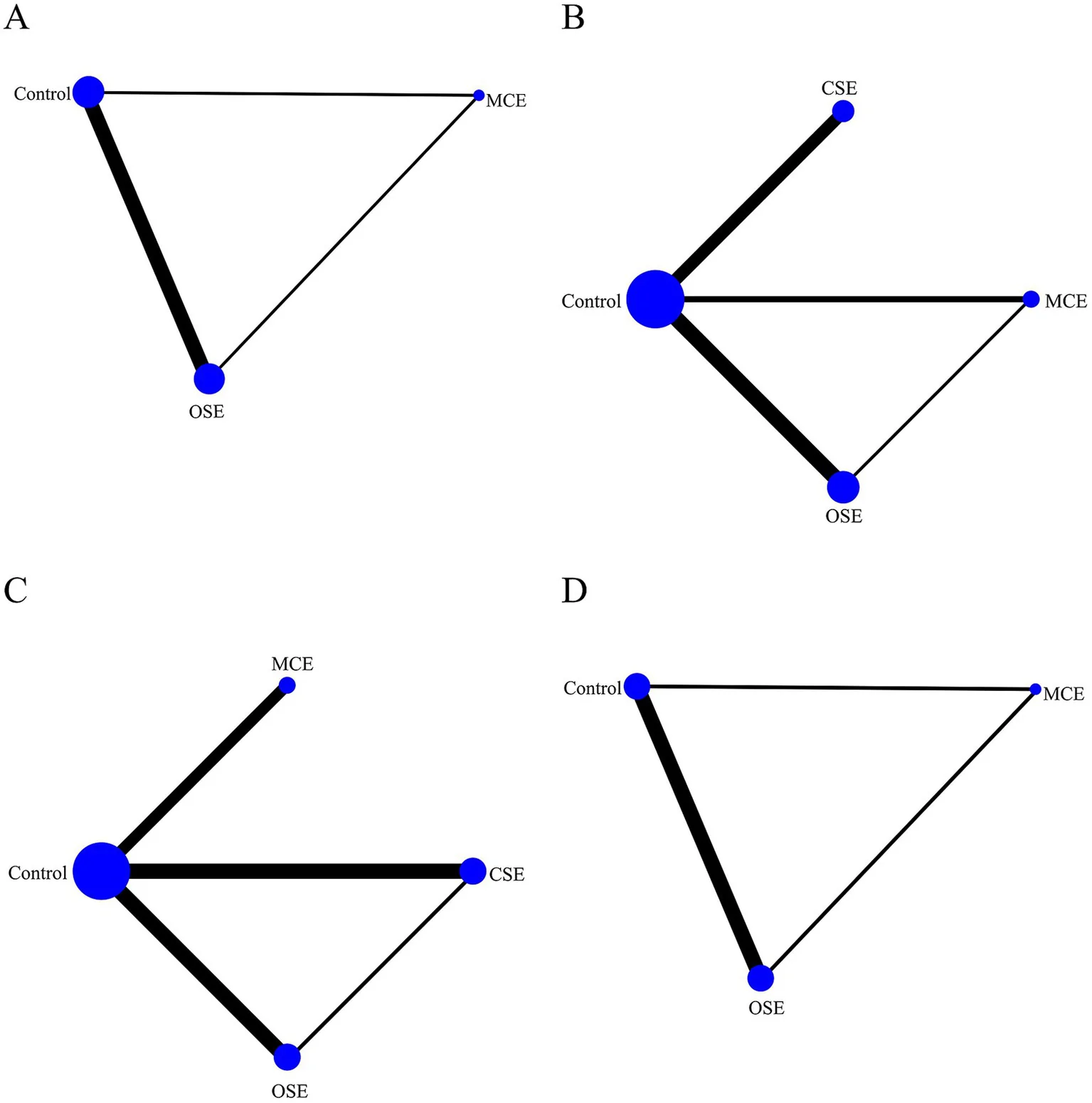
Network plots for four outcomes: **(A)** hostility; **(B)** anger; **(C)** overall aggression; **(D)** physical aggression. Node size is proportional to the number of studies involving each intervention, and line thickness is proportional to the number of studies contributing to each direct comparison. MCE, multicomponent exercise; OSE, open-skill exercise; CSE, closed-skill exercise.

**Figure 4:**
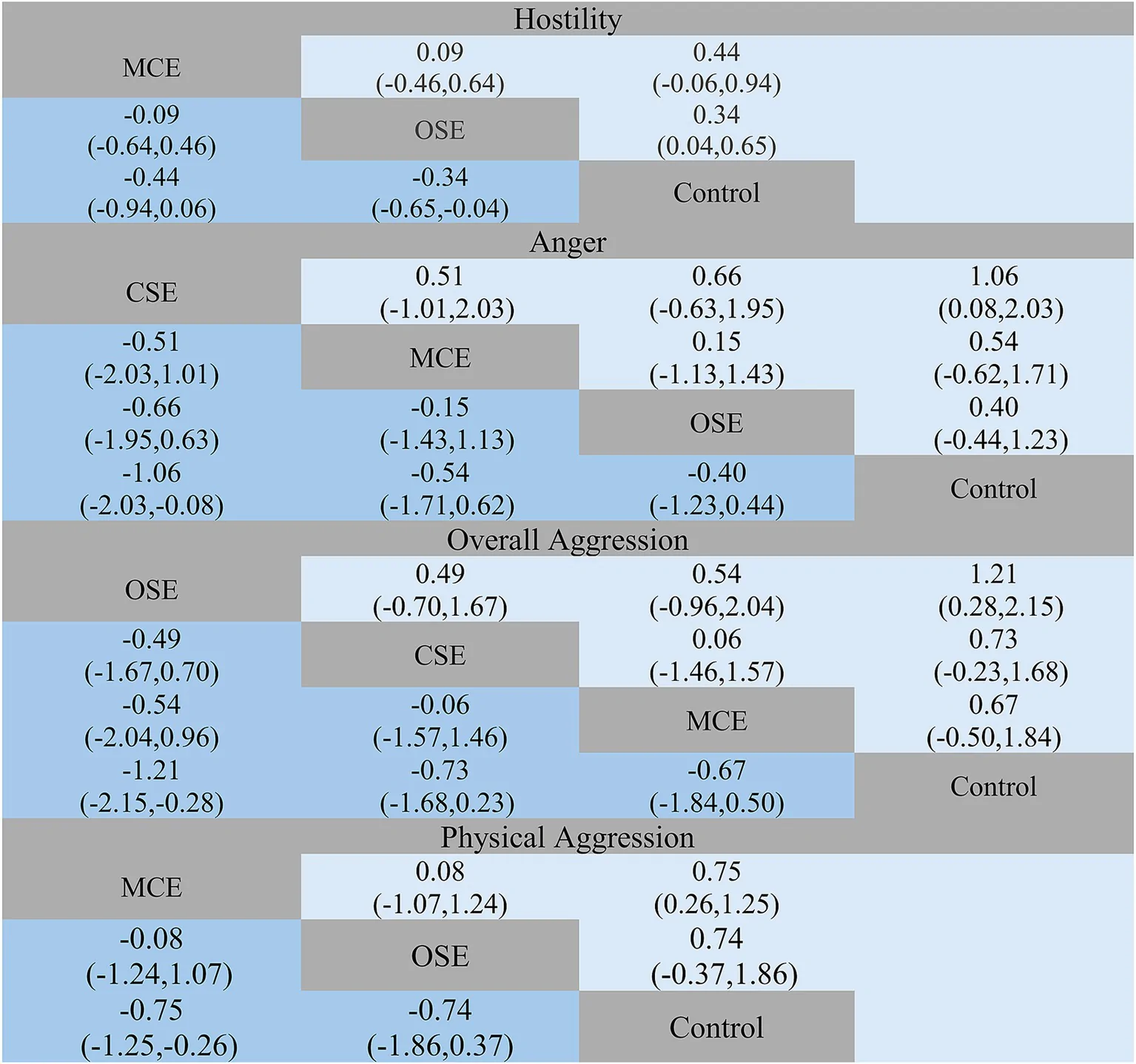
League table for comparisons between interventions. Values are presented as standardized mean differences (SMDs) with 95% confidence intervals (CIs). Each comparison represents the treatment listed in the row versus the treatment listed in the column. Positive values favor the row treatment, whereas negative values favor the column treatment. Gray diagonal cells contain intervention names that serve as both row and column headers. The lightly shaded upper triangle and blue-shaded lower triangle present the same comparisons in opposite directions. MCE, multicomponent exercise; OSE, open-skill exercise; CSE, closed-skill exercise.

**Figure 5:**
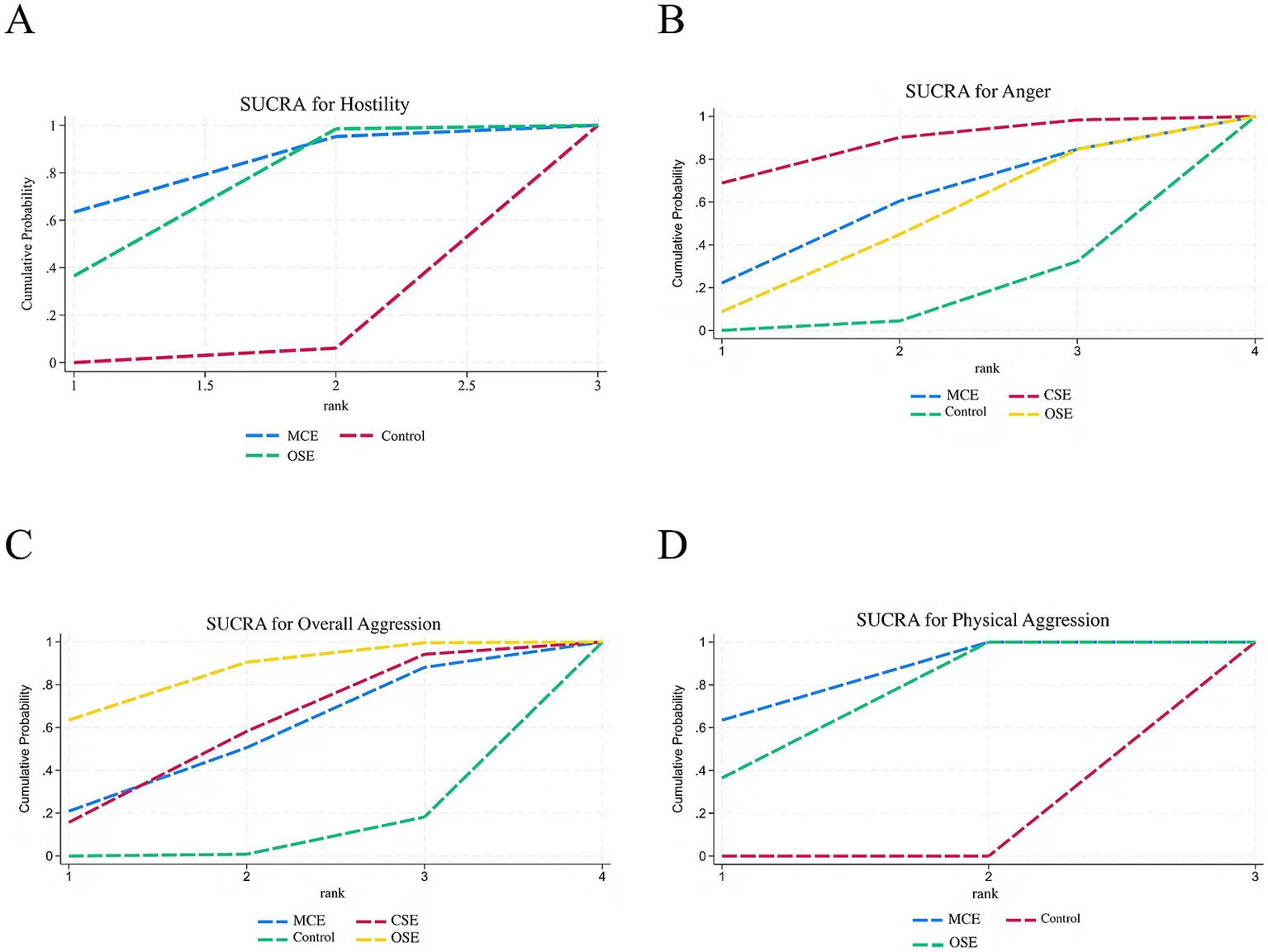
SUCRA cumulative ranking probability plot: **(A)** Hostility; **(B)** Anger; **(C)** Overall aggression; **(D)** Physical aggression. MCE, multicomponent exercise; OSE, open-skill exercise; CSE, closed-skill exercise. The horizontal axis represents the possible intervention ranks, with rank 1 indicating the most favorable rank. The vertical axis represents the cumulative probability that an intervention is ranked at a given position or better. Each curve represents an intervention or control condition. Curves that rise more steeply and remain higher at the more favorable ranks indicate higher SUCRA values and more favorable ranking distributions.

For hostility outcomes, a total of 7 studies involving 1,075 participants were included (Reynes and Lorant, 2002; Shachar et al., 2016; Trajković et al., 2020a, 2020b; Zhao et al., 2020; Zhu and Yan, 2006; Zhu F. et al., 2022; Zhu Y. et al., 2022). The exercise modalities included OSE and MCE. Five studies evaluated OSE effects against control conditions, one evaluated MCE effects against control conditions, and one compared OSE directly with MCE (Figure 3). Before conducting the network meta-analysis, conventional pairwise meta-analyses pooled data for the direct comparisons (Table 2). Compared with control conditions, exercise interventions significantly reduced hostility levels (SMD = −0.37, 95% CI: −0.62 to −0.13), despite substantial observed heterogeneity (*I^2^* = 62.5%; Supplementary Figure S1). The subsequent network meta-analysis utilized a consistency model because the global consistency test (*p* = 0.5437) and node-splitting analysis (Supplementary Table S2) indicated no significant inconsistency. League table results (Figure 4) indicated that the effects of both MCE and OSE favored reductions in hostility compared with the control condition, although only the comparison between OSE and the control condition reached statistical significance. The comparisons between MCE and the control condition and between MCE and OSE were not statistically significant. The SUCRA ranking results (Figure 5; Supplementary Table S4) showed that MCE ranked highest (79.4%), followed by OSE (67.5%). Taken together, OSE showed a statistically significant benefit compared with the control condition, whereas the ranking analysis suggested that MCE may have greater potential for reducing hostility.

**Table 2:** Results of traditional meta-analysis.

Outcome	SMD (95% CI)	*p*	*I^2^*
Hostility	−0.37(−0.62, −0.13)	<0.05	62.5%
Anger	−0.64(−1.00, −0.27)	<0.05	90.7%
Overall Aggression	−0.87(−1.36, −0.39)	<0.05	90.1%
Physical Aggression	−0.70(−0.91, −0.49)	<0.05	43.2%
Verbal Aggression	−0.05(−0.36, 0.25)	>0.05	50.2%

### 3.4 Anger

For anger outcomes, a total of 12 studies involving 1,709 participants were included (Butzer et al., 2017; Chen and Diao, 2007; He, 2007; Lok et al., 2024; Reynes and Lorant, 2002; Shachar et al., 2016; Tkacz et al., 2008; Trajković et al., 2020a, 2020b; Yang et al., 2018; Zhao et al., 2020; Zhu F. et al., 2022; Zhu Y. et al., 2022). The exercise modalities included OSE, CSE, and MCE. Five studies evaluated OSE against the control group, four evaluated CSE against the control group, two evaluated MCE against the control group, and one compared OSE with MCE (Figure 3). Pairwise meta-analysis (Table 2) showed that, compared with the control group, exercise interventions significantly reduced anger levels (SMD = −0.64, 95% CI: −1.00 to −0.27), although substantial between-study heterogeneity emerged (*I^2^* = 90.7%; Supplementary Figure S5). Because neither the global consistency test (*p* = 0.9615) nor the node-splitting analysis (Supplementary Table S7) detected significant inconsistency, a consistency model was used for the network meta-analysis. SUCRA rankings (Figure 5; Supplementary Table S9) indicated that CSE ranked highest for reducing anger (85.9%), followed by MCE (55.8%) and OSE (46.1%). The league table (Figure 4) showed that CSE significantly reduced anger compared with the control condition, whereas no statistically significant differences were observed among the active exercise interventions. These findings suggest that CSE may be a suitable exercise modality for reducing anger.

To explore potential sources of heterogeneity, meta-regression analyses were conducted using participants’ age, sample size, intervention duration, and intervention frequency as covariates. The results showed no significant associations for any evaluated variables (all *p* > 0.05). Sensitivity analyses showed that, after excluding each study one at a time, the pooled effect size ranged from SMD = −0.38 to −0.77, with no substantial change in the direction of the results. Excluding the study by Zhu F. et al. (2022); Zhu Y. et al. (2022) reduced the statistical significance of the pooled estimate to a borderline level (*p* = 0.054), suggesting that this study may have been a major source of heterogeneity. However, the direction and magnitude of the overall effect remained largely consistent with those of the primary analysis, indicating reasonable finding robustness (Supplementary Figure S7).

### 3.5 Overall aggression

For overall aggression outcomes, a total of 12 studies involving 1,004 participants were included (Fung and Lee, 2018; Guo et al., 2025; Haden et al., 2014; Harwood-Gross et al., 2021; Liang et al., 2024; Liu H. et al., 2025, Liu C. et al., 2025; Mehralian et al., 2022; Park et al., 2017; Parsamajd and Teymori, 2024; Perić et al., 2022; Velásquez et al., 2015; Wade et al., 2018). The exercise modalities included OSE, CSE, and MCE. Four studies evaluated OSE against the control group, four evaluated CSE against the control group, three evaluated MCE against the control group, and one compared OSE with CSE (Figure 3). Pairwise meta-analysis (Table 2) showed that, compared with the control group, exercise interventions significantly reduced overall aggression (SMD = −0.87, 95% CI: −1.36 to −0.39), with high between-study heterogeneity (*I^2^* = 90.1%; Supplementary Figure S11). A consistency model was adopted for the network meta-analysis because the global consistency test (*p* = 0.5792) and node-splitting analysis (Supplementary Table S12) identified no significant inconsistency. League table results (Figure 4) showed that, except for OSE, no statistically significant differences emerged between the remaining exercise interventions and the control group or among the different exercise modalities. SUCRA rankings (Figure 5; Supplementary Table S14) indicated that OSE had the highest probability of being the most effective intervention for reducing overall aggression (84.4%), followed by CSE (56.0%) and MCE (53.2%).

To explore potential sources of heterogeneity, meta-regression analyses were conducted using participants’ age, sample size, intervention frequency, intervention duration, and session length as covariates. No significant associations emerged for any of these variables (all *p* > 0.05), suggesting that these study characteristics do not represent the primary sources of heterogeneity. Sensitivity analyses showed that, after excluding each study one at a time, the pooled effect size ranged from SMD = −0.66 to −1.02. The pooled effects remained statistically significant across all models (*p* < 0.05), indicating that the robustness of the overall aggression findings (Supplementary Figure S13).

### 3.6 Physical aggression

For physical aggression outcomes, 6 studies involving 1,016 participants were included (Reynes and Lorant, 2002; Shachar et al., 2016; Trajković et al., 2020a, 2020b; Zhao et al., 2020; Zhu F. et al., 2022; Zhu Y. et al., 2022). The exercise modalities included OSE and MCE. Four studies evaluated OSE against the control group, one evaluated MCE against the control group, and one compared OSE directly with MCE (Figure 3). Conventional pairwise meta-analysis (Table 2) showed that, compared with the control group, exercise interventions significantly reduced physical aggression (SMD = −0.70, 95% CI: −0.91 to −0.49), with moderate between-study heterogeneity (*I^2^* = 43.2%; Supplementary Figure S17). Because the global consistency test detected no significant inconsistency (*p* = 0.8869), a consistency model was used for the network meta-analysis. League table results (Figure 4) showed that only MCE demonstrated a statistically significant advantage over the control group, whereas no significant differences were observed between OSE and the control group or between MCE and OSE. SUCRA rankings (Figure 5; Supplementary Table S18) indicated that MCE ranked highest for reducing physical aggression (74.7%), followed by OSE (70.9%).

### 3.7 Verbal aggression

For verbal aggression outcomes, 5 studies involving 367 participants were included (Reynes and Lorant, 2002; Trajković et al., 2020a, 2020b; Zhao et al., 2020; Zhu F. et al., 2022; Zhu Y. et al., 2022). The exercise modalities included OSE and MCE. Four studies evaluated OSE against the control group, and one compared OSE with MCE. Because evidence for MCE was limited for this outcome and failed to meet the prespecified criteria for network synthesis, no network meta-analysis was conducted. Pairwise meta-analysis (Table 2) showed that, compared with the control group, exercise interventions failed to significantly reduce verbal aggression (SMD = −0.05, 95% CI: −0.36 to 0.25). Between-study heterogeneity was moderate (*I^2^* = 50.2%; Supplementary Figure S21). Effect sizes across studies were generally small, and all 95% CIs crossed zero, suggesting that the overall effect lacked statistical significance.

### 3.8 Subgroup analyses

Subgroup analyses examined anger and overall aggression outcomes based on exercise format, specifically individual sports versus team sports. The results showed a statistically significant difference between subgroups for the anger outcome (*p* < 0.05), displaying a larger effect size for individual sports (SMD = −0.83) than for team sports (SMD = −0.50) (Supplementary Figure S6). For overall aggression, the between-subgroup difference was also statistically significant (*p* < 0.05), although team sports displayed a larger effect size (SMD = −1.08) than individual sports (SMD = −0.81) (Supplementary Figure S12). These findings suggest that exercise format may influence the intervention effects on different aggression-related outcomes. However, because heterogeneity remained high in the overall analyses and within each subgroup (*I^2^* > 50%), these results warrant cautious interpretation.

### 3.9 Meta-regression analysis

Within the network meta-analysis framework, separate meta-regression analyses evaluated the anger and overall aggression outcomes. For the anger outcome, mean participant age (*β* = 0.031, 95% CI: −0.282 to 0.344, *p* = 0.808), sample size (*β* = 0.002, 95% CI: −0.007 to 0.012, *p* = 0.549), intervention frequency (*β* = −0.307, 95% CI: −1.673 to 1.059, *p* = 0.589), intervention duration (*β* = 0.033, 95% CI: −0.078 to 0.143, *p* = 0.483), and session length (*β* = 0.005, 95% CI: −0.078 to 0.087, *p* = 0.885) did not show significant moderating effects (Supplementary Table S6).

For the overall aggression outcome, mean participant age (*β* = 0.216, 95% CI: −0.243 to 0.675, *p* = 0.280), sample size (*β* = 0.005, 95% CI: −0.014 to 0.024, *p* = 0.556), intervention frequency (*β* = 0.228, 95% CI: −1.407 to 1.864, *p* = 0.734), intervention duration (*β* = 0.029, 95% CI: −0.185 to 0.244, *p* = 0.740), and session length (*β* = 0.020, 95% CI: −0.026 to 0.066, *p* = 0.316) likewise showed no significant moderating effects. Because the 95% CIs for the regression coefficients of all covariates crossed zero, current evidence fails to confirm that these demographic or intervention characteristics explain the between-study variation in effect sizes under the present number of included studies and available data conditions (Supplementary Table S11).

## 4 Publication Bias

**Figure 6:**
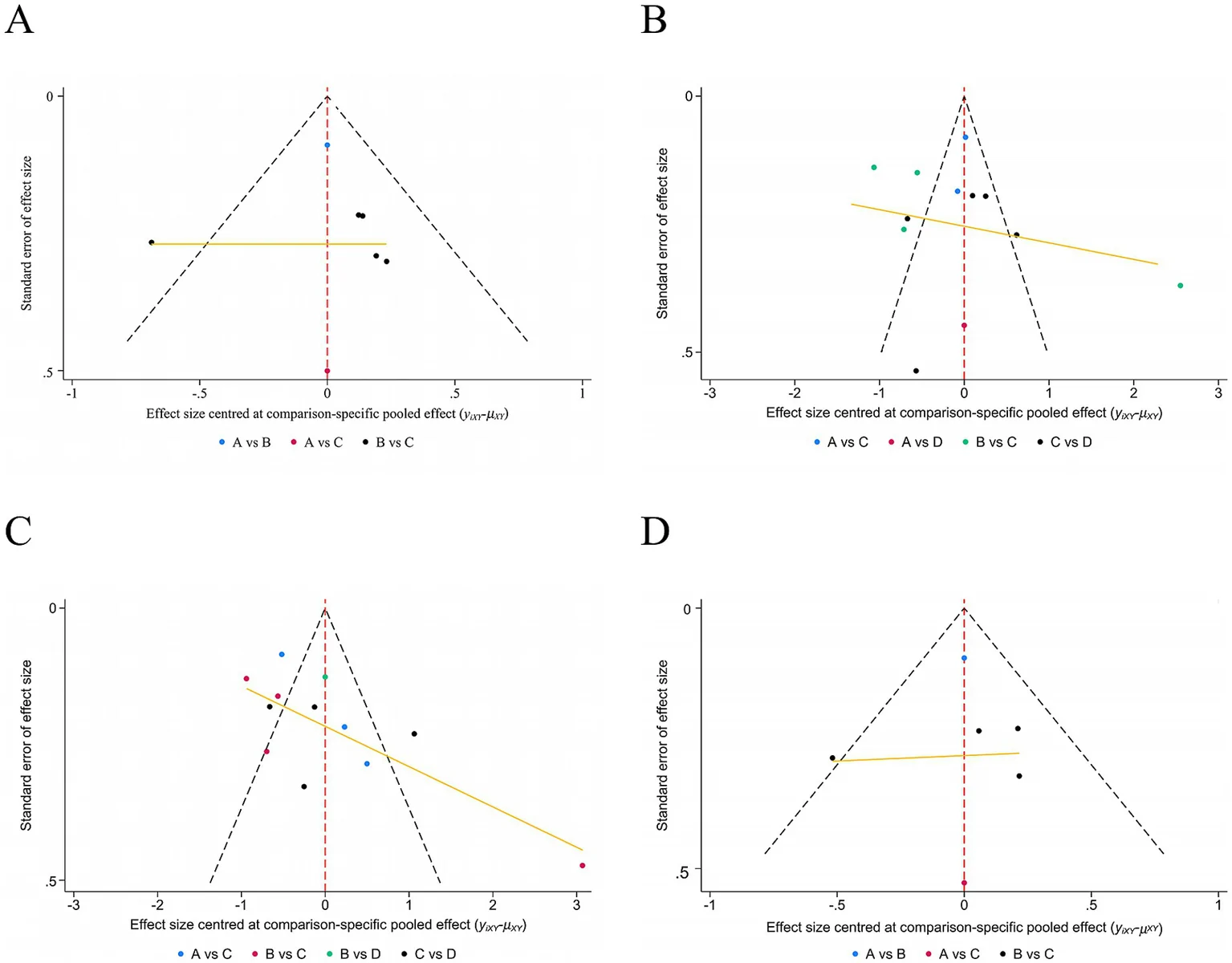
Funnel plot for publication bias: **(A)** Hostility; **(B)** Anger; **(C)** Overall aggression; **(D)** Physical aggression. In panels **(A,D)**, the treatment labels are defined as follows: A = MCE, B = Control, and C = OSE. In panels **(B,C)**, the treatment labels are defined as follows: A = MCE, B = CSE, C = Control, and D = OSE.

Funnel plots (Figure 6) and Egger’s linear regression test were used to assess funnel plot asymmetry for hostility, anger, overall aggression, and physical aggression (Egger et al., 1997; Sterne et al., 2011). Egger’s test indicated no statistically significant asymmetry for hostility (*p* = 0.2034; Supplementary Table S5), anger (*p* = 0.8856; Supplementary Table S10), or physical aggression (*p* = 0.4566; Supplementary Table S19). However, Egger’s test indicated statistically significant asymmetry for overall aggression (*p* = 0.0198; Supplementary Table S15), suggesting potential small-study effects or reporting bias. After adjustment using the nonparametric trim-and-fill method (Duval and Tweedie, 2000), the pooled effect size shifted, but the direction of the effect and the overall conclusions remained materially unaltered. This stability suggests that the overall conclusion was not materially altered after adjustment, although potential reporting bias cannot be excluded.

## 5 Discussion

This study combined conventional meta-analysis with network meta-analysis to systematically compare the relative effects of different skill-based types of physical exercise on aggression-related outcomes in children and adolescents. Overall, the results showed that exercise interventions significantly reduced hostility, anger, overall aggression, and physical aggression, whereas no significant improvement was observed for verbal aggression. This variance indicates that physical exercise primarily affects dimensions involving emotional arousal, hostile cognition, and overt behavioral control, whereas its influence on verbal aggression—which depends more heavily on language habits, social interaction patterns, and communication styles—remains uncertain.

Regarding hostility, OSE was the only exercise modality that showed a statistically significant benefit compared with the control condition, whereas MCE ranked highest in the SUCRA analysis. The difference between MCE and OSE was not statistically significant; therefore, the relative effect estimates provided clearer support for OSE, whereas MCE ranked highest in the SUCRA analysis. Hostility represents a relatively stable negative cognitive tendency (Buss and Perry, 1992) that may be difficult to modify through emotional catharsis or reduced physiological arousal alone. Its improvement may instead require repeated social interaction, rule internalization, and self-monitoring, through which individuals gradually modify their perceptions of peers and external situations (Crick and Dodge, 1994). Both OSE and MCE may support these processes, but through different intervention features. OSE may expose participants to dynamic situations that require continuous interpretation of external cues, response regulation, and behavioral adjustment, often within socially interactive and rule-governed settings (Gu et al., 2019). MCE, in contrast, combines multiple training components and provides children and adolescents with diverse physical and social contexts in which they repeatedly experience cooperation, communication, and role transitions (Eime et al., 2013). For example, Shachar et al. (2016) implemented a 24-week MCE program combining martial arts and group sports in five 50-min sessions per week. Specifically, the physical training components of MCE may help improve overall emotional states and physical self-perception (Lubans et al., 2016); cooperative or game-based activities may promote peer interaction and social connectedness (Eime et al., 2013); and rule-oriented training components may further enhance behavioral control and self-regulation through explicit task demands and continuous behavioral feedback (Diamond and Lee, 2011). Together, these components may help reduce hostility-related social-cognitive biases, including negative interpretations of peers and external situations. However, because MCE encompasses complex protocols, substantial differences may exist across studies in intervention content, intensity, duration, and the inclusion of cooperative or educational elements. The observed effects may therefore depend on the specific combination of components and the context in which the intervention is implemented.

For anger, CSE showed a statistically significant benefit compared with the control condition and ranked highest in the SUCRA analysis. However, no statistically significant differences were observed among CSE, MCE, and OSE. Thus, CSE ranked highest in the SUCRA analysis, but its apparent advantage over the other active modalities remains uncertain. At the individual-study level, significant reductions in anger were reported in aerobic exercise programs delivered five times per week for 60 min over 13 weeks (Tkacz et al., 2008) and four times per week for 60 min over 10 weeks (Lok et al., 2024). These interventions exemplify the structured, repetitive, and relatively stable training conditions characteristic of CSE. Such conditions, together with clearly defined rules and limited exposure to changing external stimuli, may provide a repeatable framework that supports self-monitoring and anger regulation. CSE also often emphasizes rhythm control and individualized practice. These characteristics may help children and adolescents redirect their attention from external conflicts or interpersonal stimuli toward bodily sensations, movement rhythms, and motor control. Over time, such training may help reduce tension and stress while improving awareness of emotional changes and the capacity for self-regulation (Lubans et al., 2016). Because anger is a high-arousal emotion that can be triggered by immediate situational cues, a low-confrontation, low-stimulation, and controllable exercise environment may help limit emotional escalation and support emotional stability. This interpretation is broadly consistent with evidence suggesting that noncontact and low-confrontation exercise contexts may be particularly suitable for anger regulation (Wang et al., 2025; Wang et al., 2025; Pels and Kleinert, 2016). This intervention profile may help explain CSE’s favorable ranking for anger reduction.

Regarding overall aggression, OSE was the only exercise modality that showed a statistically significant benefit compared with the control condition and ranked highest in the SUCRA analysis. At the individual-study level, significant reductions in overall aggression were reported following a karate program delivered three times per week for 60 min over 12 weeks (Parsamajd and Teymori, 2024), as well as soccer programs delivered twice per week for 60 min over 16 weeks (Perić et al., 2022) and three times per week for 60 min over 13 weeks (Guo et al., 2025). These examples show that OSE programs associated with reductions in overall aggression included both an individual combat sport and team-based ball sports. OSE requires individuals to continuously engage in perceptual processing, response selection, and behavioral adjustment in dynamically changing environments, thereby imposing substantial demands on inhibitory control, cognitive flexibility, and social adaptation (Ludyga et al., 2022). Existing meta-analytic evidence also suggests that OSE promotes executive function among children and adolescents (Heilmann et al., 2022). Given that overall aggression encompasses emotional, cognitive, and behavioral dimensions (Javela et al., 2023), the dynamic adaptation and behavioral regulation processes emphasized in OSE may be particularly beneficial for improving this composite outcome. Notably, the subgroup analysis provided a complementary explanation from a different classification perspective. For overall aggression, team sports showed larger effects than individual sports, suggesting that improvements in overall aggression may arise not only from the physiological effects of physical activity but also from the regulation of social behavior. Team sports involve core elements such as cooperation, competition, rule constraints, and immediate feedback. These effects may therefore reflect both exercise participation and the continuous reinforcement of behavioral boundaries, social norms, and peer interaction patterns. Team sports also provide enriched contexts for social interaction (Eime et al., 2013) and may cultivate rule awareness, role responsibility, and behavioral control through repeated collective participation (Carreres-Ponsoda et al., 2021; Opstoel et al., 2020). Conversely, individual sports yielded a larger effect size for anger, indicating that they may be particularly useful for aggression-related components driven primarily by internal regulation. Compared with team sports, individual sports emphasize autonomous regulation, self-paced control, and individualized task completion, and may reduce the immediate influence of competitive conflicts, peer evaluations, and complex social stimuli (Van Yperen, 2025; Wei et al., 2025). Their intervention effects may therefore be more apparent in the regulation of emotional arousal and recovery processes associated with anger.

Although the intervention examples described above provide some practical context, the current evidence is insufficient to establish a clear dose–response relationship. For both anger and overall aggression, meta-regression analyses found no significant moderating effects of intervention frequency, intervention duration, or session duration. The interventions in these studies were typically delivered two to five times per week, with each session lasting 50–60 min, over 10–24 weeks. Their planned total exercise time ranged from approximately 32 to 100 h. However, these ranges merely reflect the characteristics of the included intervention protocols and should not be interpreted as a minimum effective dose, an optimal dose, or a definitive dosing recommendation. They should instead be viewed only as examples of programs associated with beneficial outcomes.

For physical and verbal aggression, the intervention effects of physical exercise showed different patterns. For physical aggression, MCE ranked highest in the SUCRA analysis and was the only exercise modality showing a statistically significant benefit compared with the control conditions. Because physical aggression represents an overt behavioral manifestation, its reduction may involve emotion regulation paired with enhanced behavioral restraint and inhibitory control (Kyranides et al., 2024). Therefore, MCE’s favorable ranking may be related partly to its multimodal training characteristics. Within a single protocol, MCE simultaneously involves physical activity, motor control, rule adherence, task switching, and interactive practice, thereby imposing high cognitive engagement and strict behavioral regulation demands on children and adolescents. Previous research suggests that cognitively engaging physical activity may improve executive function, particularly inhibitory control, in children and adolescents (Teng et al., 2025). By contrast, verbal aggression exhibited no significant improvement. Unlike physical aggression, verbal aggression remains deeply embedded in daily communication and social interaction contexts. Its development and maintenance stem from emotional impulsivity, peer norms, and the broader school social environment (Laninga-Wijnen et al., 2021; Li et al., 2021). Therefore, although physical exercise may improve negative emotions and impulse control, these changes may not necessarily translate directly into mature forms of expression or healthy patterns of conflict communication. Existing meta-analytic evidence also indicates that exercise interventions yield limited or nonsignificant effects on verbal aggression in children and adolescents (Zhao and Wang, 2025). This limitation suggests that future interventions targeting verbal aggression may need to combine physical exercise with relevant psychosocial intervention components.

In summary, physical exercise represents a promising nonpharmacological strategy to improve aggression-related outcomes in children and adolescents, although its efficacy varies across different dimensions of aggression. Different exercise modalities show different outcome-specific ranking patterns; however, these differences manifest primarily within network comparisons and ranking probabilities. Therefore, the findings of this study are more appropriately interpreted as providing preliminary evidence to inform, rather than definitive guidance for, the targeted selection of exercise interventions.

### 5.1 Limitations

This study has several limitations. First, the small number of trials reporting verbal aggression yielded insufficient direct comparative data, preventing the establishment of a connected evidence network. Therefore, the synthesis relied solely on direct evidence pooling rather than a complete network meta-analysis. Second, substantial statistical heterogeneity emerged across several outcomes. Although sensitivity analyses evaluated eligible parameters and network meta-regression explored potential heterogeneity sources, only a limited number of outcomes met the necessary volume requirements for meta-regression. Corresponding analyses could not be performed for the remaining outcomes; therefore, the sources of heterogeneity could not be fully explained (Cuijpers et al., 2021). Third, although this study used SMDs to synthesize results across different scales to minimize the influence of measurement differences, inconsistencies in the assessment tools for aggression-related outcomes and in the sources of informant reports may still have affected comparability across studies (De Los Reyes and Kazdin, 2005). In addition, the CINeMA assessment indicated that all network comparisons were rated as having low or very low certainty, mainly because of within-study bias, imprecision, and heterogeneity.

Missing data were present at both the trial and review levels. At the trial level, 21 studies were rated as having a low risk of bias for incomplete outcome data, whereas four were rated as having an unclear risk (Harwood-Gross et al., 2021; Liu H. et al., 2025, Liu C. et al., 2025; Shachar et al., 2016; Trajković et al., 2020a). This indicates that some studies did not adequately report or address participant attrition or missing outcome data, which may have increased the risk of bias in the effect estimates. At the review level, some studies did not fully report the statistical information required to calculate effect sizes, thereby limiting the amount of data available for quantitative synthesis.

The risk-of-bias assessment also identified methodological limitations in the included studies. Of the 25 studies, 24 were rated as having an unclear risk of bias for allocation concealment, 23 as having an unclear risk for blinding of outcome assessors, and 23 as having a high risk for blinding of participants and personnel. Because the content of exercise interventions and group assignments is often apparent, blinding participants and personnel can be difficult in exercise trials. Nevertheless, the lack of blinding may increase the risk of performance bias and expectation effects. Fung and Lee (2018) reduced this risk to some extent by using an active physical fitness control condition and blinded cluster allocation. Liu H. et al., (2025); Liu C. et al., (2025) reported that allocation was conducted by an independent researcher and that outcome assessors were blinded, but information on blinding of participants and intervention personnel remained insufficient. These methodological limitations should be considered when interpreting the pooled effect estimates.

The geographic distribution of the included evidence was also uneven. Of the 25 studies, 16 were conducted in Asia, none in Africa, only one in South America, and four in Europe, three of which were conducted in Serbia. The concentration of evidence in a small number of countries and research settings may limit the generalizability of the findings across populations with different cultural backgrounds, educational systems, and exercise delivery contexts. In addition, although four major databases were searched, relevant studies indexed only in other specialized databases or not formally published may have been missed.

### 5.2 Implications for practice, policy, and future research

For generally healthy children and adolescents in educational settings, structured exercise may be incorporated as a component of universal or selective prevention programs to promote emotion regulation, behavioral control, and prosocial interaction. However, the specific exercise modality should be selected according to the targeted behavioral outcome and the practical conditions of the school. For adolescents with diagnosed behavioral disorders, clinically significant aggression, or attention-deficit/hyperactivity disorder (ADHD), exercise may serve as an adjunct to psychological, educational, and medical interventions, but it should not replace diagnostic evaluation or established psychological, behavioral, educational, or pharmacological treatment. Exercise programs for adolescents with clinical concerns should be individually tailored according to symptom severity, medication use, physical capacity, risk of interpersonal conflict, and capacity for sustained participation, with coordination among families, schools, exercise professionals, and healthcare providers.

At the policy level, schools and youth-serving organizations may consider integrating structured exercise programs into routine physical education and behavioral support services while ensuring that students with different needs have opportunities to participate. Adolescents with evident behavioral or psychological difficulties should receive timely referral for further psychological assessment and professional support, and collaboration between physical education teachers and mental health professionals should be strengthened. Current evidence is insufficient to support a uniform recommendation regarding exercise modality and dose for all children and adolescents. Further research is needed in general-population, at-risk, and clinical samples, particularly among adolescents with ADHD and disruptive behavior disorders. More specifically, research should prioritize areas where the current evidence remains weak. For outcomes such as verbal aggression, which feature scarce data and lack a connected evidence network, investigators must conduct additional high-quality RCTs to improve the robustness of relative effect estimates and the credibility of intervention rankings (Nikolakopoulou et al., 2020; Salanti et al., 2022). Furthermore, given that distinct exercise modalities exert differential effects on specific aggression dimensions, future studies should clarify the relative efficacy of OSE, CSE, and MCE via adequately powered head-to-head comparisons. In addition, future research should investigate potential mechanisms of action and key effect modifiers while standardizing outcome measurement and reporting to enhance the comparability and interpretability of future evidence syntheses.

## 6 Conclusion

Physical exercise interventions significantly mitigate hostility, anger, overall aggression, and physical aggression in children and adolescents. Conversely, current empirical evidence fails to confirm a clear intervention effect of physical exercise on verbal aggression. The network meta-analysis showed that MCE may have potential to reduce hostility and physical aggression, CSE may be useful for mitigating anger, and OSE may provide benefits for decreasing overall aggression. Structured exercise may therefore be incorporated into school-based prevention programs for generally healthy children and adolescents. For adolescents with clinically significant behavioral problems or ADHD, exercise may serve an adjunctive role; however, the current evidence remains insufficient to support diagnosis-specific recommendations.

## Data Availability

The raw data supporting the conclusions of this article will be made available by the authors, without undue reservation.
