# Supplementary material for: Effects of different types of physical exercise interventions on aggressive behavior among children and adolescents: a network meta-analysis
Source: Front Psychol. 2026 Sep 9;17:1927181. doi: 10.3389/fpsyg.2026.1927181 (PMC13597820; doi:10.3389/fpsyg.2026.1927181)
Supplement: Supplementary file 1 [file Supplementary_file_1.DOCX]

**Supplementary Material-1**

**Contents**: This supplementary material includes the complete search strategy, definitions of key concepts used in the network meta-analysis, and additional analyses, figures, and tables for the outcomes of hostility, anger, overall aggression, physical aggression, and verbal aggression. The materials include CINeMA evidence assessment, pairwise meta-analysis results, subgroup analyses, meta-regression analyses, sensitivity analyses, node-splitting analyses, ranking probabilities, SUCRA values, ranking probability plots, cumulative ranking probability plots, network meta-analysis forest plots, and publication bias tests.

**Supplementary Data 1. Search strategy**

**PubMed**

("Child"[Mesh] OR "Adolescent"[Mesh] OR child*[tiab] OR children[tiab] OR adolescen*[tiab] OR teen*[tiab] OR youth*[tiab] OR juvenile*[tiab] OR pediatric*[tiab] OR paediatric*[tiab] OR schoolchild*[tiab]) AND ("Exercise"[Mesh] OR "Exercise Therapy"[Mesh] OR "Sports"[Mesh] OR "Motor Activity"[Mesh] OR exercise*[tiab] OR "physical exercise"[tiab] OR "physical activity"[tiab] OR "physical activities"[tiab] OR sport*[tiab] OR training[tiab] OR "exercise intervention*"[tiab] OR "physical activity intervention*"[tiab] OR "sport intervention*"[tiab] OR aerobic[tiab] OR "aerobic exercise"[tiab] OR resistance[tiab] OR "resistance training"[tiab] OR "strength training"[tiab] OR "martial art*"[tiab] OR yoga[tiab] OR dance[tiab] OR "team sport*"[tiab] OR "individual sport*"[tiab] OR "physical education"[tiab]) AND ("Aggression"[Mesh] OR "Violence"[Mesh] OR "Bullying"[Mesh] OR aggress*[tiab] OR "aggressive behavior"[tiab] OR "aggressive behaviour"[tiab] OR violen*[tiab] OR hostility[tiab] OR anger[tiab] OR bully*[tiab] OR "conduct problem*"[tiab] OR "externalizing behavior"[tiab] OR "externalising behaviour"[tiab] OR "antisocial behavior"[tiab] OR "antisocial behaviour"[tiab])

**Web of Science**

TS=( (child* OR children OR adolescen* OR teen* OR youth* OR juvenile* OR pediatric* OR paediatric* OR schoolchild* ) AND ( "physical exercise" OR exercise* OR "physical activity" OR "physical activities" OR sport* OR training OR "exercise intervention*" OR "physical activity intervention*" OR "sport intervention*" OR aerobic OR "aerobic exercise" OR resistance OR "resistance training" OR "strength training" OR "martial art*" OR yoga OR dance OR "team sport*" OR "individual sport*" OR "physical education" ) AND ( aggress* OR "aggressive behavior" OR "aggressive behaviour" OR violen* OR hostility OR anger OR bully* OR "conduct problem*" OR "externalizing behavior" OR "externalising behaviour" OR "antisocial behavior" OR "antisocial behaviour" ))

**Embase**

('child'/exp OR 'adolescent'/exp OR child*:ti,ab,kw OR children:ti,ab,kw OR adolescen*:ti,ab,kw OR teen*:ti,ab,kw OR youth*:ti,ab,kw OR juvenile*:ti,ab,kw OR pediatric*:ti,ab,kw OR paediatric*:ti,ab,kw OR schoolchild*:ti,ab,kw) AND ('exercise'/exp OR 'exercise therapy'/exp OR 'sport'/exp OR 'physical activity'/exp OR exercise*:ti,ab,kw OR 'physical exercise':ti,ab,kw OR 'physical activity':ti,ab,kw OR 'physical activities':ti,ab,kw OR sport*:ti,ab,kw OR training:ti,ab,kw OR 'exercise intervention*':ti,ab,kw OR 'physical activity intervention*':ti,ab,kw OR 'sport intervention*':ti,ab,kw OR aerobic:ti,ab,kw OR 'aerobic exercise':ti,ab,kw OR resistance:ti,ab,kw OR 'resistance training':ti,ab,kw OR 'strength training':ti,ab,kw OR 'martial art*':ti,ab,kw OR yoga:ti,ab,kw OR dance:ti,ab,kw OR 'team sport*':ti,ab,kw OR 'individual sport*':ti,ab,kw OR 'physical education':ti,ab,kw) AND ('aggression'/exp OR 'violence'/exp OR 'bullying'/exp OR aggress*:ti,ab,kw OR 'aggressive behavior':ti,ab,kw OR 'aggressive behaviour':ti,ab,kw OR violen*:ti,ab,kw OR hostility:ti,ab,kw OR anger:ti,ab,kw OR bully*:ti,ab,kw OR 'conduct problem*':ti,ab,kw OR 'externalizing behavior':ti,ab,kw OR 'externalising behaviour':ti,ab,kw OR 'antisocial behavior':ti,ab,kw OR 'antisocial behaviour':ti,ab,kw)

**CNKI**

(儿童 OR 青少年 OR 少年 OR 中学生 OR 小学生 OR 学生 OR 学龄儿童 OR 学校儿童)AND(运动 OR 体育运动 OR 身体活动 OR 体育锻炼 OR 体育活动 OR 运动干预 OR 身体活动干预 OR 体育干预 OR 运动训练 OR 体育训练 OR 有氧运动 OR 抗阻训练 OR 力量训练 OR 武术 OR 瑜伽 OR 舞蹈 OR 团队运动 OR 集体运动 OR 个人运动 OR 个体运动 OR 体育教育 OR 体育课)AND(攻击 OR 攻击性 OR 攻击行为 OR 攻击性行为 OR 暴力 OR 校园暴力 OR 欺凌 OR 校园欺凌 OR 同伴欺凌 OR 同伴攻击 OR 身体攻击 OR 言语攻击 OR 关系攻击 OR 敌意 OR 愤怒 OR 品行问题 OR 外化行为 OR 反社会行为)

**Supplementary Data 2. Stata code**

1.The standardized mean difference (SMD) was used as the summary effect size for continuous outcomes measured on different scales across studies: network setup mean sd n, study(id) trt(t) format(augment) smd

2.Network Graph Generation: network map, improve

3.Inconsistency Model Testing: network meta i

4.Analysis Using the Consistency Model: network meta c

5.node-splitting method: network sidesplit all, tau

6.Calculation of SUCRA for Each Intervention (Lower MD Indicates Better Rank) : network rank min, all zero reps(5000) gen(prob)

7.Larger effect sizes indicate better ranks: network rank max, all zero reps(5000) gen(prob)

8.Cumulative Rankogram: sucra prob*, lab(1 2 3 4 5 6 7 8 9 10 11)

9.Ranking Probability Plot: sucra prob*, lab(1 2 3 4 5 6 7 8 9 10 11) rankog

10.League table: netleague, lab(1 2 3 4 5 6 7 8 9 10 11) sort(11 10 9 8 7 6 5 4 3 2 1)

11.Forest Plots of Pairwise Comparisons: intervalplot, null(0) lab(1 2 3 4 5 6 7 8 9 10 11)

12.Funnel Plot: netfunnel _y _stderr _t1 _t2 , random bycomp add(lfit _stderr _ES_CEN) noalpha

**Supplementary Note S1. Definitions of Key Concepts Used in the Network Meta-analysis**

**Node-splitting**

A method that separates the direct and indirect evidence for a specific treatment comparison and evaluates whether the two sources of evidence are statistically consistent.

**SUCRA**

A summary measure of the cumulative ranking probabilities of each intervention, ranging from 0% to 100%. A higher SUCRA value indicates a greater probability that an intervention ranks among the more effective options.

**Direct evidence**

Evidence obtained from studies that directly compare two interventions within the same trial.

**Indirect evidence**

Evidence used to estimate the relative effects of two interventions through one or more common comparators when no direct comparison is available.

**Network structure**

The arrangement and connectivity of interventions and direct comparisons within a network meta-analysis. In a network plot, nodes represent interventions, whereas connecting lines represent available direct comparisons.

**local inconsistency**

Disagreement between direct and indirect evidence for a specific treatment comparison or part of the evidence network.

**Supplementary Table S1. Evidence assessment using CINeMA**

1. **Hostility**

**Supplementary Figure S1. Forest plot of pairwise meta-analysis for hostility**

**Supplementary Table S2. Node-splitting analysis for hostility**

| **Side** | **Direct Coef.** | **Direct Std. Err.** | **Indirect Coef.** | **Indirect Std. Err.** | **Difference Coef.** | **Difference Std. Err.** | **P>\|z\|** | **tau** |
| --- | --- | --- | --- | --- | --- | --- | --- | --- |
| A B | .5204371 | .3076942 | .130394 | .5632448 | .3900431 | .6418103 | 0.543 | .2971544 |
| A C | -.1885372 | .5379531 | .2013457 | .3506112 | -.3898829 | .6421228 | 0.544 | .2971542 |
| B C | -.3188931 | .1682674 | -.7090568 | .6197185 | .3901638 | .6421565 | 0.543 | .2971787 |

**Supplementary Table S3. Ranking probabilities for hostility**

| **Rank** | **MCE** | **Control** | **OSE** |
| --- | --- | --- | --- |
| **Best** | 63.4 | 0.0 | 36.5 |
| **2nd** | 31.9 | 6.1 | 62.0 |
| **Worst** | 4.7 | 93.9 | 1.4 |

**Supplementary Figure S2. Cumulative ranking probability plot for hostility**

**Supplementary Figure S3. Rank probability plot for hostility**

**Supplementary Table S4. SUCRA values and ranking results for hostility**

| **Treatment** | **SUCRA** | **PrBest** | **MeanRank** |
| --- | --- | --- | --- |
| MCE | 79.4 | 63.4 | 1.4 |
| Control | 3.1 | 0.0 | 2.9 |
| OSE | 67.5 | 36.5 | 1.6 |

**Supplementary Figure S4. Forest plot of network meta-analysis estimates for hostility**

**Supplementary Table S5. Egger’s regression test for publication bias in hostility**

| **Test Type** | **Model** | **β₁ / Kendall's Score** | **Standard Error (SE)** | **z-Value** | **p-Value** |
| --- | --- | --- | --- | --- | --- |
| Egger's Regression | Random-effects (REML) | 2.37 | 1.867 | 1.27 | 0.2034 |

2. **Anger**

**Supplementary Figure S5. Forest plot of pairwise meta-analysis for anger**

**Supplementary Figure S6. Forest plot of subgroup analysis for anger**

**Supplementary Table S6. Meta-regression analysis for anger**

| **Covariate** | **Coefficient** | **Std. err.** | **t** | **P>\|t\|** | **[95% conf. interval]** |
| --- | --- | --- | --- | --- | --- |
| **Age** | .0312842 | .1217833 | 0.26 | 0.808 | -.2817698 to .3443382 |
| **Sample size** | .0024058 | .0037429 | 0.64 | 0.549 | -.0072157 to .0120273 |
| **Intervention frequency** | -.3068397 | .5314588 | -0.58 | 0.589 | -1.672998 to 1.059319 |
| **Intervention period** | .0325377 | .0429915 | 0.76 | 0.483 | -.0779755 to .143051 |
| **Intervention duration** | .0048682 | .0320433 | 0.15 | 0.885 | -.0775017 to .087238 |
| **Constant** | -1.620993 | 3.404178 | -0.48 | 0.654 | -10.37171 to 7.129727 |

**Supplementary Figure S7. Sensitivity analysis for anger**

**Supplementary Table S7. Node-splitting analysis for anger**

| **Side** | **Direct Coef.** | **Direct Std. Err.** | **Indirect Coef.** | **Indirect Std. Err.** | **Difference Coef.** | **Difference Std. Err.** | **P>\|z\|** | **tau** |
| --- | --- | --- | --- | --- | --- | --- | --- | --- |
| A C | .563154 | .735189 | .4942898 | 1.22125 | .0688642 | 1.425468 | 0.961 | 1.029914 |
| A D | .1045393 | 1.122972 | .1733716 | .8780993 | -.0688324 | 1.425526 | 0.961 | 1.029918 |
| B C | 1.056379 | .4991353 | 1.04495 | 315.8652 | .0114291 | 315.8656 | 1.000 | .966348 |
| C D | -.3897696 | .4801865 | -.4586197 | 1.342222 | .0688502 | 1.425533 | 0.961 | 1.029919 |

**Supplementary Table S8. Ranking probabilities for anger**

| **Rank** | **MCE** | **CSE** | **Control** | **OSE** |
| --- | --- | --- | --- | --- |
| **Best** | 22.2 | 68.9 | 0.1 | 8.8 |
| **2nd** | 38.3 | 21.3 | 4.4 | 36.1 |
| **3rd** | 24.2 | 8.2 | 27.8 | 39.7 |
| **Worst** | 15.3 | 1.6 | 67.7 | 15.4 |

**Supplementary Figure S8. Cumulative ranking probability plot for anger**

**Supplementary Figure S9. Rank probability plot for anger**

**Supplementary Table S9. SUCRA values and ranking results for anger**

| **Treatment** | **SUCRA** | **PrBest** | **MeanRank** |
| --- | --- | --- | --- |
| MCE | 55.8 | 22.2 | 2.3 |
| CSE | 85.9 | 68.9 | 1.4 |
| Control | 12.3 | 0.1 | 3.6 |
| OSE | 46.1 | 8.8 | 2.6 |

**Supplementary Figure S10. Forest plot of network meta-analysis estimates for anger**

**Supplementary Table S10. Egger’s regression test for publication bias in anger**

| **Test Type** | **Model** | **β₁ / Kendall's Score** | **Standard Error (SE)** | **z-Value** | **p-Value** |
| --- | --- | --- | --- | --- | --- |
| Egger's Regression | Random-effects (REML) | -0.42 | 2.924 | -0.14 | 0.8856 |

3 **Overall Aggression**

**Supplementary Figure S11. Forest plot of pairwise meta-analysis for overall aggression**

**Supplementary Figure S12. Forest plot of subgroup analysis for overall aggression**

**Supplementary Table S11. Meta-regression analysis for overall aggression**

| **Covariate** | **Coefficient** | **Std. err.** | **t** | **P>\|t\|** | **[95% conf. interval]** |
| --- | --- | --- | --- | --- | --- |
| **Age** | .2163646 | .1785838 | 1.21 | 0.280 | -.2426996 to .6754288 |
| **Sample size** | .0046885 | .007436 | 0.63 | 0.556 | -.0144265 to .0238034 |
| **Intervention frequency** | .2283633 | .636145 | 0.36 | 0.734 | -1.4069 to 1.863626 |
| **Intervention period** | .0292447 | .0834228 | 0.35 | 0.740 | -.1852006 to .2436899 |
| **Intervention duration** | .0199111 | .0178831 | 1.11 | 0.316 | -.026059 to .0658811 |
| **Constant** | -6.05904 | 2.856974 | -2.12 | 0.087 | -13.40313 to 1.285047 |

**Supplementary Figure S13. Sensitivity analysis for overall aggression**

**Supplementary Table S12. Node-splitting analysis for overall aggression**

| **Side** | **Direct Coef.** | **Direct Std. Err.** | **Indirect Coef.** | **Indirect Std. Err.** | **Difference Coef.** | **Difference Std. Err.** | **P>\|z\|** | **tau** |
| --- | --- | --- | --- | --- | --- | --- | --- | --- |
| A C | .6703749 | .5992107 | .5384718 | 105.3711 | .1319031 | 105.3728 | 0.999 | .9960935 |
| B C | .8485874 | .5791494 | .2788175 | 1.232662 | .5697699 | 1.362009 | 0.676 | 1.081497 |
| B D | -.8393837 | 1.095657 | -.2695949 | .8090995 | -.5697888 | 1.362023 | 0.676 | 1.081498 |
| C D | -1.118183 | .5648251 | -1.687961 | 1.23929 | .5697784 | 1.362009 | 0.676 | 1.081496 |

**Supplementary Table S13. Ranking probabilities for overall aggression**

| **Rank** | **MCE** | **CSE** | **Control** | **OSE** |
| --- | --- | --- | --- | --- |
| **Best** | 20.9 | 15.7 | 0.0 | 63.5 |
| **2nd** | 29.7 | 42.5 | 0.8 | 27.0 |
| **3rd** | 37.5 | 36.0 | 17.4 | 9.1 |
| **Worst** | 11.9 | 5.9 | 81.7 | 0.5 |

**Supplementary Figure S14. Cumulative ranking probability plot for overall aggression**

**Supplementary Figure S15. Rank probability plot for overall aggression**

**Supplementary Table S14. SUCRA values and ranking results for overall aggression**

| **Treatment** | **SUCRA** | **PrBest** | **MeanRank** |
| --- | --- | --- | --- |
| MCE | 53.2 | 20.9 | 2.4 |
| CSE | 56.0 | 15.7 | 2.3 |
| Control | 6.4 | 0.0 | 3.8 |
| OSE | 84.4 | 63.4 | 1.5 |

**Supplementary Figure S16. Forest plot of network meta-analysis estimates for overall aggression**

**Supplementary Table S15. Egger’s regression test for publication bias in overall aggression**

| **Test Type** | **Model** | **β₁ / Kendall's Score** | **Standard Error (SE)** | **z-Value** | **p-Value** |
| --- | --- | --- | --- | --- | --- |
| Egger's Regression | Random-effects (REML) | -6.34 | 2.721 | -2.33 | 0.0198 |

**4 physical Aggression**

**Supplementary Figure S17. Forest plot of pairwise meta-analysis for physical aggression**

**Supplementary Table S16. Node-splitting analysis for physical aggression**

| **Side** | **Direct Coef.** | **Direct Std. Err.** | **Indirect Coef.** | **Indirect Std. Err.** | **Difference Coef.** | **Difference Std. Err.** | **P>\|z\|** | **tau** |
| --- | --- | --- | --- | --- | --- | --- | --- | --- |
| A B | .752358 | .2532507 | .6678754 | .5333891 | .0844825 | .5904573 | 0.886 | .2398587 |
| A C | .00807 | .5074794 | .0920521 | .3022955 | -.0839821 | .5906928 | 0.887 | .2398612 |
| B C | -.6601749 | .1651324 | -.7444357 | .567145 | .0842609 | .5906964 | 0.887 | .2398803 |

**Supplementary Table S17. Ranking probabilities for physical aggression**

| **Rank** | **MCE** | **Control** | **OSE** |
| --- | --- | --- | --- |
| **Best** | 63.50 | 0.00 | 36.50 |
| **2nd** | 36.50 | 0.00 | 63.50 |
| **Worst** | 0.00 | 100.00 | 0.00 |

**Supplementary Figure S18. Cumulative ranking probability plot for physical aggression**

**Supplementary Figure S19. Rank probability plot for physical aggression**

**Supplementary Table S18. SUCRA values and ranking results for physical aggression**

| **Treatment** | **SUCRA** | **PrBest** | **MeanRank** |
| --- | --- | --- | --- |
| MCE | 74.7 | 63.5 | 1.5 |
| Control | 4.4 | 0.0 | 2.9 |
| OSE | 70.9 | 36.5 | 1.6 |

**Supplementary Figure S20. Forest plot of network meta-analysis estimates for physical aggression**

**Supplementary Table S19. Egger’s regression test for publication bias in physical aggression**

| **Test Type** | **Model** | **β₁ / Kendall's Score** | **Standard Error (SE)** | **z-Value** | **p-Value** |
| --- | --- | --- | --- | --- | --- |
| Egger's Regression | Random-effects (REML) | 2.12 | 2.842 | 0.74 | 0.4566 |

**5 Verbal Aggression**

**Supplementary Figure S21. Forest plot of pairwise meta-analysis for verbal aggression**

**Supplementary Figure S22. Network meta-analysis forest plots and SUCRA rankings**

(A)hostility; (B) anger; (C) overall aggression; (D)physical aggression.
